# Spermine Alleviates Drought Stress in White Clover with Different Resistance by Influencing Carbohydrate Metabolism and Dehydrins Synthesis

**DOI:** 10.1371/journal.pone.0120708

**Published:** 2015-04-02

**Authors:** Zhou Li, Wen Jing, Yan Peng, Xin Quan Zhang, Xiao Ma, Lin Kai Huang, Yan-hong Yan

**Affiliations:** Department of Grassland Science, College of Animal Science and Technology, Sichuan Agricultural University, Chengdu, 611130, China; Louisiana State University Agricultural Center, UNITED STATES

## Abstract

The objective of this research was to analyse whether ameliorating drought stress through exogenously applied spermine (Spm) was related to carbohydrate metabolism, dehydrins accumulation and the transcription of genes encoding dehydrins in two white clovers (drought-susceptible cv. ‘Ladino’ and drought-resistant cv. ‘Haifa’) under controlled drying conditions for 10 days. The results show that the application of Spm effectively alleviates negative effects caused by drought stress in both cultivars. Exogenous Spm led to accumulation of more water-soluble carbohydrates (WSC), sucrose, fructose and sorbitol in both cultivars under drought stress, and also significantly elevated glucose content in leaves of drought-resistant cv. ‘Haifa’, but had no effect on drought-susceptible cv. ‘Ladino’. Accordingly, the key enzyme activities of sucrose and sorbitol metabolism changed along with the application of Spm under drought stress. Spm induced a significant increase in sucrose phosphate synthase (SPS) or sorbitol dehydrogenase (SDH) activity, but decrease in sucrose synthetase (SS) activity when two cultivars were subjected to drought. In addition, the improved accumulation of dehydrins induced by exogenous Spm coincided with three genes expression which was responsible for dehydrins synthesis. But Spm-induced transcript level of dehydrin genes increased earlier in cv. ‘Ladino’ than that in cv. ‘Haifa’. Thus, these results suggest that ameliorating drought stress through exogenously applied Spm may be associated with increased carbohydrate accumulation and dehydrins synthesis. There are differences between drought-susceptible and -resistant white clover cultivars related to Spm regulation of WSC metabolism and dehydrins expression.

## Introduction

Water deprivation is considered as one of the most significant limitative factors in the process of plant growth and development [1]. In response to drought stress, the accumulation of water soluble carbohydrates, together with other compatible solutes such as proline, is widely regarded as an adaption for plants to maintain leaf cell turgor, since osmotic potential of plant cells was easy to be affected by drought stress [2,3]. However, carbohydrate metabolites not only play important roles in osmotic adjustments and osmoprotectants, but also act as energy supply and metabolite signaling molecules which modulate the transcript level of genes involved in drought tolerance [4]. It has been reported that enzymes of carbohydrate metabolism corresponded with numbers of stress responsive genes in *Arabidopsis* under salt, cold and drought stresses [5]. Different sugars, such as sucrose, fructose, and glucose, each has their own unique function in response to drought stress. The mulriple function and types of carbohydrates complicate the analysis of mechanisms related to drought tolerance in plants.

Sorbitol, which belongs to the main sugar alcohols or polyols, is produced in both shoot tips and mature leaves of plants [6]. Increased transport of sorbitol occurs frequently as a result of drought stress [7]. In parallel with sucrose, though sorbitol has similar function of providing translocation of carbon and energy source, it plays a major role in osmotic adjustment related to sucrose [8]. Numerous studies have found that more than 50% of total osmotic adjustment was attributed to the accumulation of sorbitol induced by drought stress [9,10]. It has been reported that sorbitol and glucose were kept at higher levels in leaves of young apple seedling whereas sucrose declined gradually during drought stress [6]. In fact, ongoing interaction between carbohydrate and sugar alcohols existed in stressed plants and their metabolism and transportation could not be isolated when plants are subjected to drought stress.

Multifactorial traits react to drought stress or dehydration including changes of protein synthesis and degradation. The accumulation of dehydrins was observed in many plant species in response to drought [11,12]. These proteins, known as late embryogenesis abundant (LEA) proteins, were highly conserved in plants (consensus sequence EKKGIMDKIKEKLPG), and were involved in protecting cellular structures, maintaining the stabilization of membrane and regulating the cell osmotic potential under drought stress [13–15]. Because of functions of inhibiting the coagulation of macromolecules and extreme hydrophilicity, dehydrins supplemented the protection afforded by sucrose accumulation [16]. Shen et al. [17] found that the overexpression of dehydrins *DcDh2* improved the tolerance of tobacco to water stress. Wang et al. [18] observed that exogenous abscisic acid (ABA) induced expression of dehydrins associated with improved drought tolerance in orchid protocorms. Although lots of previous studies have approved that dehydrins were associated with drought tolerance of plants, but there is not a clear understanding of regulation of dehydrins by other phytohormones such as polyamines (PAs) under drought stress.

As an aliphatic amine, PAs including putrescine (Put), spermidine (Spd), and spermine (Spm) occupy fundamental roles in regulating growth and development as well as stress tolerance in plants. It has been revealed that the protective functions of PAs are involved in scavenging free radical, regulating osmotic potential and proline metabolism under abiotic stress [19–21]. Although most of PAs have similar effects on improving stress tolerance in plants, Spm seems to be the most effective among PAs [22]. Exogenous application of Spm promoted acclimation to osmotic stress in soybean associated with modifying antioxidant and jasmonic acid signal [23]. Sagor et al. [24] demonstrated that Spm protected *Arabidopsis* from heat damage via involvement in elevating transcript level of heat shock-related genes; however, the protective effect was induced by Spd to a lesser extent but not by Put.

Molecular and biochemical responses of PAs to drought have been reported in many plant species [25–27]. However, limited researches have focused on PAs regulation of carbohydrate metabolism and dehydrins synthesis under drought stress. The main objectives of this study were (i) to analyse whether ameliorating drought stress through exogenously applied Spm was relate to carbohydrate and sorbitol metabolism; (ii) to assess the effects of exogenous Spm on dehydrins accumulation and genes expression in drought-resistant and drought-susceptible white clover cultivars under drought stress. Such research will further expand insight on function of PAs in improving drought tolerance in plants.

## Materials and Methods

### Plant materials and treatments

White clover seeds cv. ‘Haifa’ (tolerant to drought) and cv. ‘Ladino’ (sensitive to drought) were sown in plates which filled with sterilized quartz sand after surface-sterilizing in 0.1% mercuric chloride for 4 min and then rinsing 3 times with ddH_2_O. Plates were placed in the growth chamber (12 h photoperiod, 75% relative humidity, and 23/19°C day/night temperature). After 7 d of germination in ddH_2_O, the seedlings of white clover were watered by Hoagland’s solution [28] for another 23 d. Four treatments (four biological replicates for each treatment) were set up for drought stress: 1) L: cv. ‘Ladino’ was treated by 20% PEG 6000 Hoagland’s solution (W/V); 2) L+Spm: cv. ‘Ladino’ was treated by 20% PEG 6000 (W/V) Hoagland’s solution containing 0.5 mM spermine; 3) H: cv. ‘Haifa’ was treated by 20% PEG 6000 Hoagland’s solution (W/V); 4) H+Spm: cv. ‘Haifa’ was treated by 20% PEG 6000 (W/V) Hoagland’s solution containing 0.5 mM spermine. Before drought stress, L+Spm and H+Spm treatments were pretreated by Hoagland’s solution containing 0.5 mM Spm for 7 d in order to make white clover plants absorb enough Spm. The concentration of Spm and PEG were chosen based on a preliminary test for the obvious effects on phenotypic changes. The leaves were sampled at 0, 5 and 10 d under drought stress.

### Determination of physiological parameters

The formula RWC (%) = [(FW—DW)/(TW—DW)]×100 was used for determining leaf relative water content (fresh weight (FW), dry weight (DW), and turgid weight (TW)). Fresh leaves were weighed for FW. Leaves were then immersed in deionized water for 24 h at 4°C. After gently blotted, leaves were weighed for TW. Finally, samples were dried in an oven (80°C) for 72 h for DW [29]. Total chlorophyll content was extracted in mixture of 80% acetone and 95% ethanol (1:1, v/v) in the dark for 72 h, and then leaf extract was measured by using a spectrophotometer at 663 and 645 nm [30]. Malondialdehyde (MDA) and electrolyte leakage (EL) were determined according to methods of Dhindsa et al. [31] and Blum and Ebercon [32], respectively.

### Determination of carbohydrates and sorbitol content

Water-soluble carbohydrates (WSC) were quantified according to the method of Robyt and White [33] with modification. 0.05 g dry weight of leaves was extracted in 2 ml 80% (v/v) methanol in an 80°C water bath for 40 min. The extract was then centrifuged at 5000 rpm for 10 min to get the supernatant for the estimation of WSC. The reaction mixture was heated to 100°C in a water bath for 10 min (1 ml supernatant, 4 ml 98% sulphuric acid and 1 ml 5% phenol). The cooled reaction mixture was determined at 490 nm using D-glucose as standard. For reducing sugars (glucose and fructose) quantification, the procedure was conducted following the method used by Fu and Dernoeden [34]. 0.05 g dry tissue was extracted in 1 ml of 92% ethanol. After centrifuging at 15000 rpm for 10 min, 1 ml aliquot of supernatant was combined with 1.25 ml ferricyanide reagent and placed in a boiled water bath for 10 min. After cooling tubes to room temperature, 2.5 ml of 2 N H_2_SO_4_ was added. The absorbance of the intermixture was measured at 515 nm using a spectrophotometer based on a glucose or fructose standard curve as described by Ting [35]. For determination of sucrose, a 2 ml aliquot of supernatant was incubated in 2 ml of 4% H_2_SO_4_ (w/v) in a boiling water bath for 15 min to hydrolyze sucrose into reducing sugars and then was neutralized with 1 ml of 1 N NaOH. Ferricyanide and arsenomolybdate reagents were added to each test tube as described previously, and the absorbance was determined at 515 nm. Sorbitol analysis was carried out by using spectrophotometric method. A blue complex can form when sorbitol in alkaline solution mix with copper ions and this blue complex has an absorption peak at 655 nm. The kit (catalogue no.SY8) for the analysis was supplied by Suzhou Keming science and technology co., Ltd. (China).

### Enzymes activities of carbohydrates metabolism

Sucrose synthase (SS) and sucrose phosphate synthase (SPS) activities were determined by using the method described in Khayat and Naftaly [36] and Fu et al. [37]. For the extraction of enzymes, 0.2 g samples were ground in 5 ml of solution containing 50 mM Hepes-NaOH buffer (pH 7.5), 1 mM EDTA, 0.5 mM MgCl_2_, 2 mM diethyldithiocarbamic acid, 2% polyvinylpyrrolidone, 1% bovine serum albumin, and 2.5 mM dithiothreitol. After centrifuging for 20 min at 12000 rmp, the supernatant was collected and added into reaction solution to analyze the activity of SPS and SS. The reaction solution for the SS activity included glucose and fructose as a substrate. The SPS reaction solution was composed of 1 mM UDP-glucose, 1 mM fructose, 0.5 mM MgCl_2_, 0.5 mM NaF, 0.5 mM Na_2_MoO_4_, and 1 mM Hepes-NaOH buffer (pH 7.5). The blank reaction solution did not contain UDP-glucose. 1 ml of 1 N NaOH was added to stop the reaction. The activity of SPS and SS was measured using the resorcinol colorimetric method. Sorbitol dehydrogenase (SDH) activity was measured by observing the change of mixture absorbency (0.2 ml desalted extract, 0.1 M Tris-HCl buffer, and 1 mM NAD^+^ and 400 mM sorbitol) on a spectrophotometer at 340 nm [38].

### Western blot analysis of drought stress proteins

Soluble proteins were extracted from 0.5 g leaves of white clover in ice cold 100 mM Tris–HCl buffer (pH 8.0) and then centrifuged at 15000 rmp for 10 min (4°C). The supernatant was collected and boiled for 10 min. After recentrifuging at 12000 rmp, the sediment (an equal amount of 30 μg proteins) was used for determination of heat-stable protein and dehydrins. The BioRad mini protean transblotter was used for transferring SDS-PAGE (12%) to PVDF membranes. After 1 h of transference at 4°C and 100 V, the membranes were blocked in TRIS-buffered saline for 1 h [39,40]. Remove the TRIS-buffered saline and then wash the blots briefly in TTBS for 3 times (each 5 min). The washed membranes were incubated in rabbit anti-dehydrins dilution (1:1000) for 1 h against the conserved consensus sequence K segment (TGEKKGIMDKIKEKLPGQH) of dehydrins. After that, the membranes were rinsed in TTBS for 3 times (each 5 min) and incubated in dilution of goat anti-rabbit IgG antibody (1:2000) as the second antibody for 1 h. After washing in TTBS for 20 min, the dehydrins bands were detected by using the TMB reagent kit (Sigma) [41].

### Total RNA extraction and qRT-PCR analyses

Transcript levels of dehydrin genes were performed using a real-time quantitative polymerase chain reaction (qRT-PCR). For total RNA, 0.1 g fresh leaves of white clover were extracted by using RNeasy Mini Kit (Qiagen) according the instructions. A revert Aid First Stand cDNA Synthesis Kit (Fermentas) was used for reverse-transcribing RNA to cDNA. The cDNA was subjected to qPCR using primers of Y_2_SK, Y_2_K, SK_2_ [40] and *β*-Actin as internal control (Table 1). Transcript levels of each gene were measured using an iCycler iQ qRT-PCR detection system with SYBR Green Supermix (Bio-Rad). Four biological replicates with independent cDNA preparations were tested in this study. The conditions of the PCR protocol for all genes (Y_2_SK, Y_2_K, SK_2_ and *β*-Actin) were as follows: 5 min at 94°C and denaturation at 95°C for 30 s (40 repeats), annealing at 58°C (SK_2_ and *β*-Actin) or 60°C (Y_2_SK and Y_2_K) for 30 s and extension at 72°C for 30 s. At the end of PCR cycle, the transcript level of all genes was calculated according to the formula 2^-ΔΔCt^ described by Xia et al. [42].

**Table 1:** Primer sequences and their corresponding GeneBank accession numbers of the analyzed genes.

Target gene	Accession no.	Forward primer (5'-3')	Reverse primer (5'-3')
Dehydrin, Y_2_SK	GU443965.1	GTGCGATGGAGATGCTGTTTG	CCTAATCCAACTTCAGGTTCAGC
DHN1, Y_2_K	JF748410.1	AGCCACGCAACAAGGTTCTAA	TTGAGGATACGGGATGGGTG
Dhn b, SK_2_	GU443960.1	TGGAACAGGAGTAACAACAGGTGGA	TGCCAGTTGAGAAAGTTGAGGTTGT
*β*-Actin	JF968419	TTACAATGAATTGCGTGTTG	AGAGGACAGCCTGAATGG

### Statistical analysis

The data was analyzed by using SAS 9.1 (SAS Institute, Cary, NC). The significant relationships among treatments are based on differences between means at *P* ≤ 0.05.

## Results

### Phenotypic response and physiological analysis

**Fig 1:**
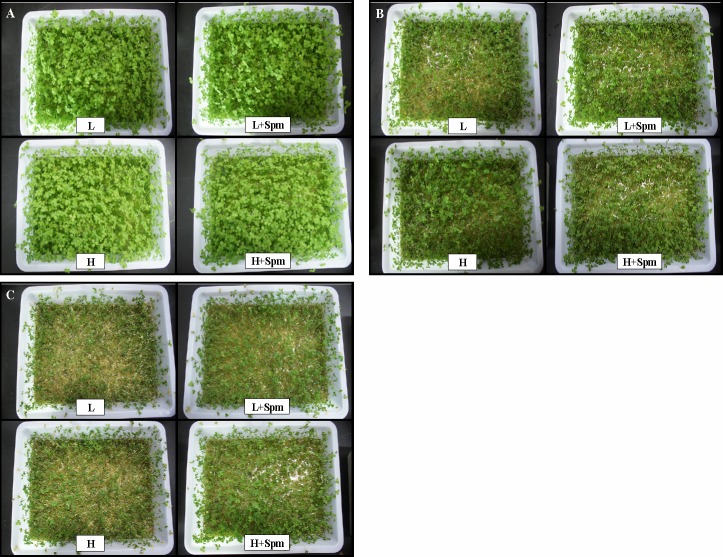
The Phenotypic responses in white clover under drought stress. (A) 0 days of drought stress, (B) 5 days of drought stress, and (C) 10 days of drought stress. L, cv. ‘Ladino’; L+Spm, cv. ‘Ladino’ added exogenous Spm; H, cv. ‘Haifa’, H+Spm, cv. ‘Haifa’ added exogenous Spm.

**Fig 2:**
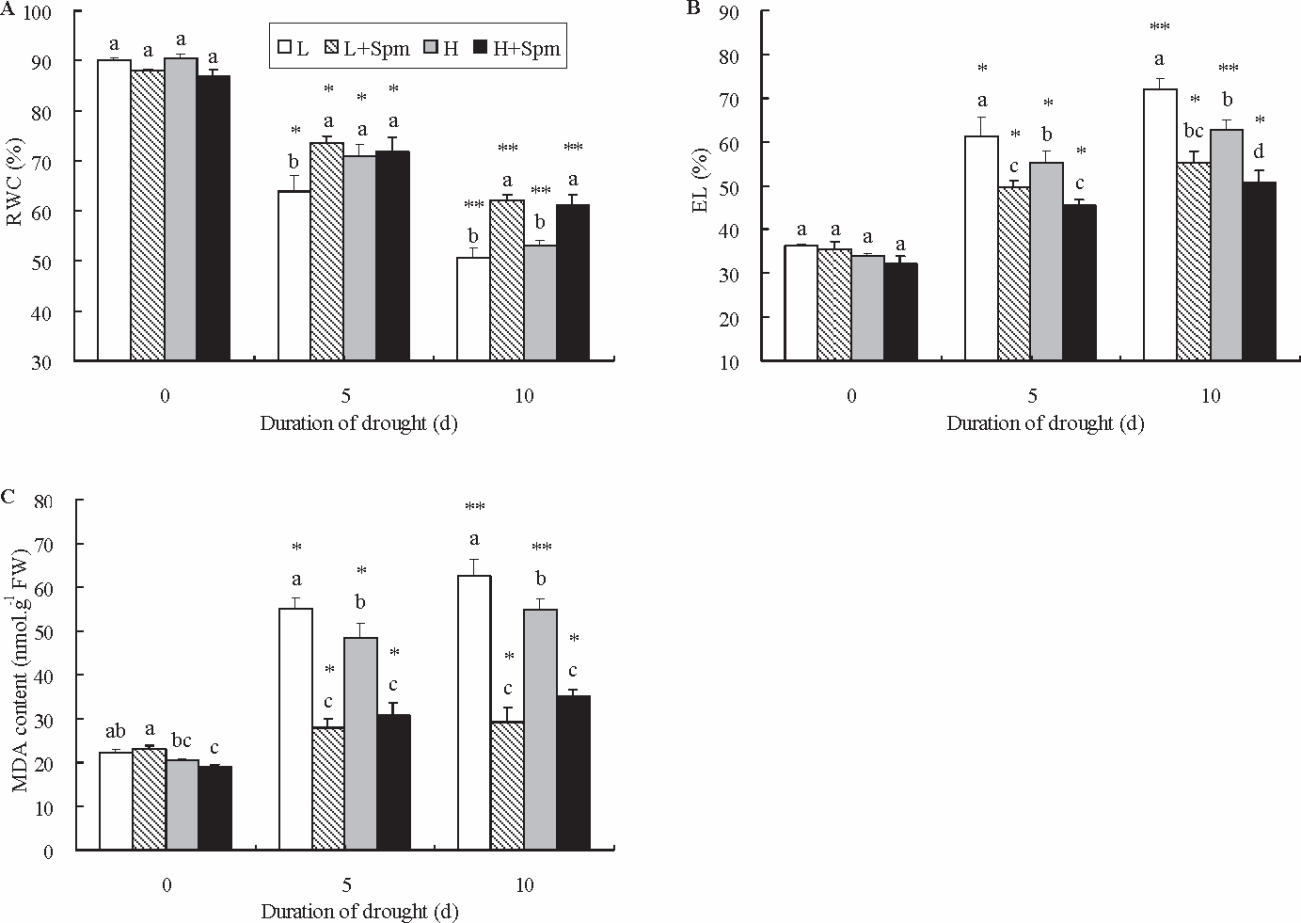
The effect of exogenous spermine (Spm) on (A) relative water content (RWC), (B) electrolyte leakage (EL) and (C) malondialdehyde (MDA) content in leaves of white clover under drought stress. Vertical bars indicate ±SE of mean (n = 4). Different letters above columns indicate significant difference for comparison at a given day; the same number of asterisks above columns means significant differences for a particular treatment across days of drought stress don’t exist. LSD (*P*≤0.05). L, cv. ‘Ladino’; L+Spm, cv. ‘Ladino’ added exogenous Spm; H, cv. ‘Haifa’, H+Spm, cv. ‘Haifa’ added exogenous Spm.

**Fig 3:**
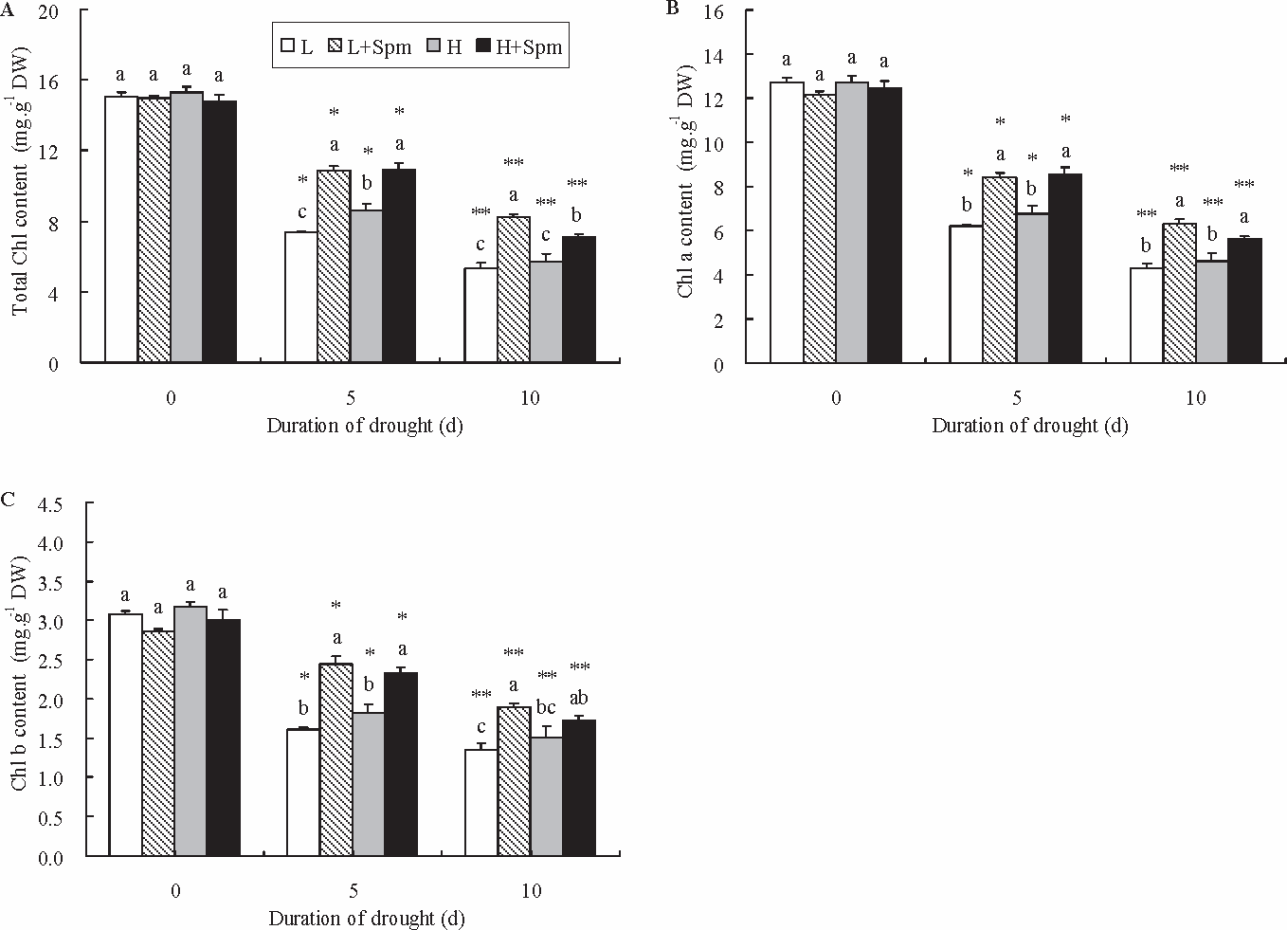
The effect of exogenous spermine (Spm) on (A) total chlorophyll, (B) chlorophyll a and (C) chlorophyll b content in leaves of white clover under drought stress. Vertical bars indicate ±SE of mean (n = 4). Different letters above columns indicate significant difference for comparison at a given day; the same number of asterisks above columns means significant differences for a particular treatment across days of drought stress don’t exist. LSD (*P*≤0.05). L, cv. ‘Ladino’; L+Spm, cv. ‘Ladino’ added exogenous Spm; H, cv. ‘Haifa’, H+Spm, cv. ‘Haifa’ added exogenous Spm.

Phenotypic responses of two white clover cultivars under drought stress were showed as Fig. 1. Spm-treated plants (L+Spm or H+Spm) stayed green and turgid as compared to untreated plants (L or H) under the same duration of drought stress. Leaf RWC was not significantly different among four treatments at the initiation of drought stress. RWC decreased gradually in response to drought stress in both cultivars, but RWC in ‘L+Spm’ treatment was maintained at a significantly higher level than that in ‘L’ treatment at 5 and 10 d of drought stress. At the last day of drought stress, ‘H+Spm’ treatment also showed a 24% higher RWC than ‘H’ treatment with a measurable significant difference (Fig. 2A). Drought stress caused steep rise of EL and MDA content in leaves of both cultivars, but exogenous Spm effectively reduced the increase trend of EL and MDA (Fig. 2B,C). During drought stress, ‘H’ showed significant lower EL and MDA content as compared to ‘L’. At 5 and 10 d of drought stress, EL level in ‘L’ or ‘H’ was 1.2 times greater than that in ‘L+Spm’ or ‘H+Spm’; on the contrary, ‘L+Spm’ or ‘H+Spm’ exhibited 100% or 55% lower MDA content than ‘L’ or ‘H’ at 5 and 10 d of drought stress, respectively, which showed significant differences (Fig. 2B,C). Progressive drought stress induced significant decline of Chl content in white clover cultivars (Fig. 3). Chl a content of Spm-treated plants (‘L+Spm’ or ‘H+Spm’) was significantly higher than that of non-treated plants (‘L’ or ‘H’) under drought stress, and Chl b content was 53% and 40% percent higher in ‘L+Spm’ than that in ‘L’ at 5 and 10 d of drought stress, respectively; this percentage was only 28% and 14% for ‘H+Spm’ comparing with ‘H’ (Fig. 3B,C). As a result, Spm-treated plants showed significantly higher total Chl content throughout the drought stress as compared to non-treated plants (Fig. 3A).

### Carbohydrates and activities of SS and SPS

**Fig 4:**
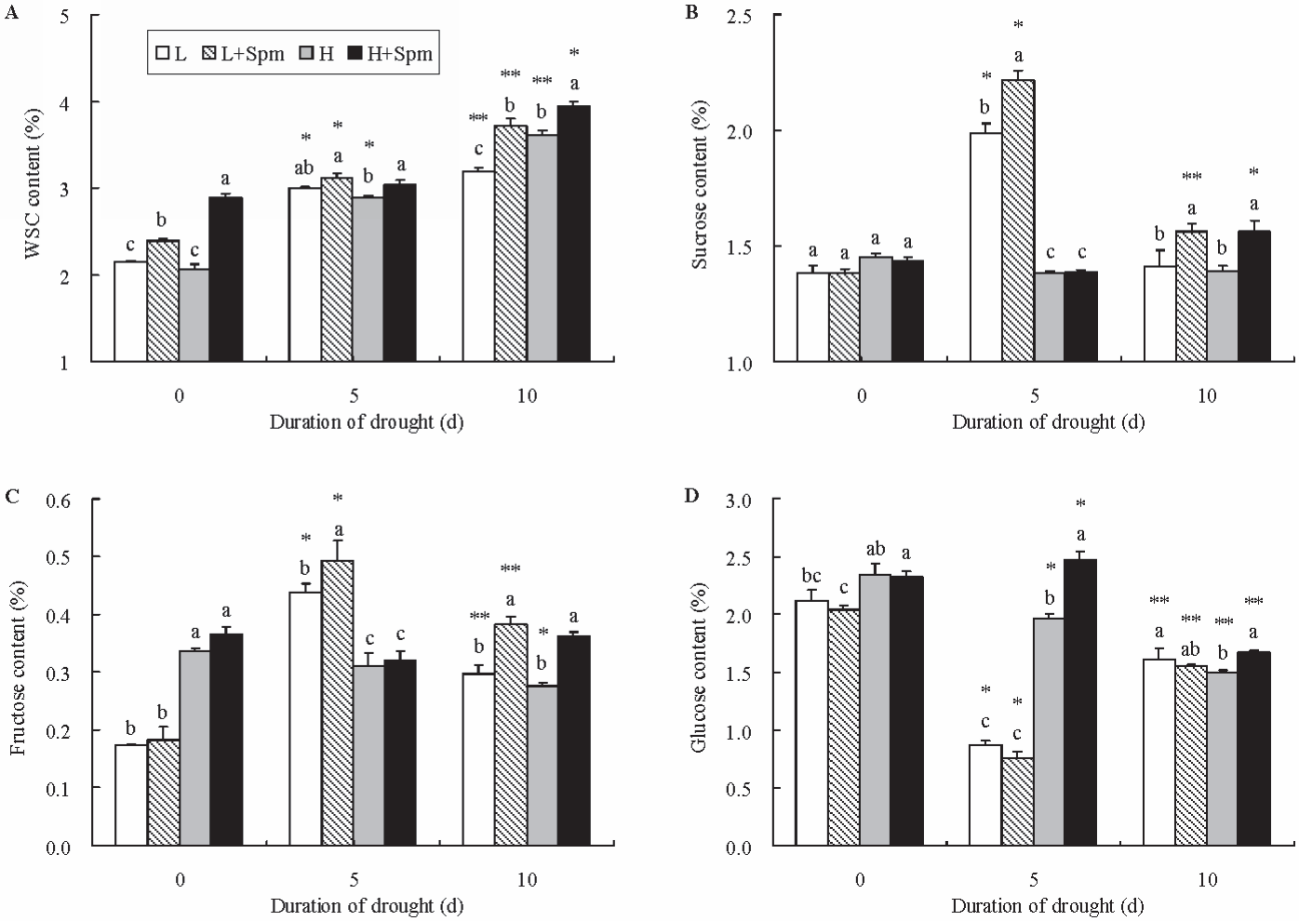
The effect of exogenous spermine (Spm) on (A) water-soluble carbohydrates (WSC), (B) sucrose, (C) fructose, and (D) glucose content in leaves of white clover under drought stress. Vertical bars indicate ±SE of mean (n = 4). Different letters above columns indicate significant difference for comparison at a given day; the same number of asterisks above columns means significant differences for a particular treatment across days of drought stress don’t exist. LSD (*P*≤0.05). L, cv. ‘Ladino’; L+Spm, cv. ‘Ladino’ added exogenous Spm; H, cv. ‘Haifa’, H+Spm, cv. ‘Haifa’ added exogenous Spm.

Drought stress strongly affected the accumulation of WSC in white clover leaves and content of WSC in four treatments reached to maximum after 10 d of drought stress. Compared to ‘L’ treatment, ‘L+Spm’ had 17% percent higher WSC content at the end of drought stress. ‘H+Spm’ showed significantly higher WSC content than ‘H’ treatment throughout the whole process of drought stress (Fig. 4A). In addition, sucrose contents of ‘L’ and ‘L+Spm’ treatment displayed a ~ 2-fold and ~ 3-fold increase after 5 d of drought, respectively. When drought stress lasted for 5 and 10 d, ‘L+Spm’ accumulated significantly higher sucrose content relative to ‘L’. With the development of drought stress, little change of sucrose content in cv. ‘Haifa’ was observed, while ‘H+Spm’ exhibited significantly higher sucrose content than ‘H’ at the end of drought stress (Fig. 4B). Plants of cv. ‘Haifa’ had significantly higher fructose content than plants of cv. ‘Ladino’ at the beginning. Although without statistically significant difference in fructose between ‘H+Spm’ and ‘H’ at 5 d of drought stress, ‘H+Spm’ displayed significantly higher fructose level after 10 d of water deficit relative to ‘H’ treatment. Fructose content in ‘L+Spm’ also maintained a significantly higher level under 5 and 10 d of drought stress as compared to that in ‘L’ (Fig. 4C). Exogenous Spm-treatment almost had no effect on glucose accumulation in cv. ‘Ladino’ under drought stress. However, glucose content in ‘H+Spm’ was considerably greater than that in ‘H’ under drought stress, and a peak value was visible in ‘H+Spm’ treatment at 5 d of stress (Fig. 4D).

**Fig 5:**
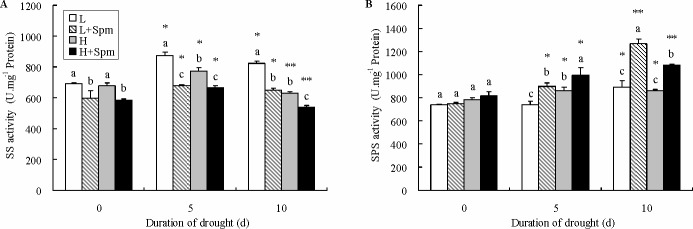
The effect of exogenous spermine (Spm) on (A) sucrose synthetase (SS) and (B) sucrose phosphate synthase (SPS) activity in leaves of white clover under drought stress. Vertical bars indicate ±SE of mean (n = 4). Different letters above columns indicate significant difference for comparison at a given day; the same number of asterisks above columns means significant differences for a particular treatment across days of drought stress don’t exist. LSD (*P*≤0.05). L, cv. ‘Ladino’; L+Spm, cv. ‘Ladino’ added exogenous Spm; H, cv. ‘Haifa’, H+Spm, cv. ‘Haifa’ added exogenous Spm.

Exogenous Spm-treatment in both cultivars decreased SS activity from 0 to 10 d of drought stress. This decreased percentage was 13%, 23% and 22% in cv. ‘Ladino’ at 0, 5, and 10 d of treatment, respectively, and 13%, 14% and 14% in cv. ‘Haifa’, correspondingly (Fig. 5A). SPS activity was relatively unchanged or slightly increased in non-treated plants (‘H’ or ‘L’) during drought stress, but obviously increased by 69% or 33% in ‘L+Spm’ or ‘H+Spm’ treatment after 10 d of drought stress. Accordingly, significant differences of SPS activities between Spm-treated plants (‘L+Spm’ or ‘H+Spm’) and non-treated plants (‘L’ or ‘H’) were observed at 5 and 10 d of drought stress (Fig. 5B).

### Sorbitol content and SDH activity

**Fig 6:**
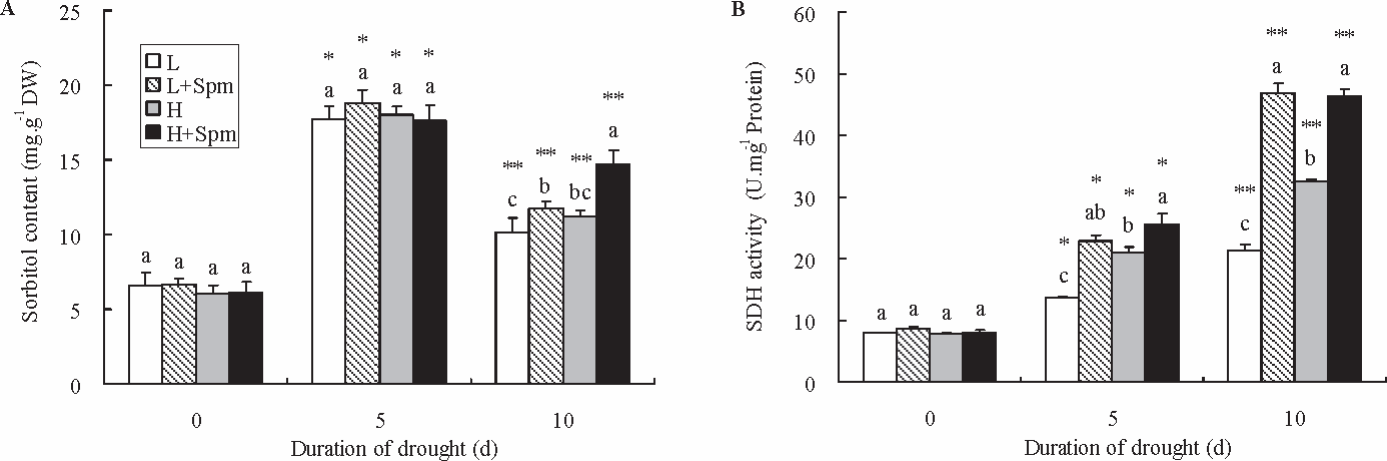
The effect of exogenous spermine (Spm) on (A) sorbitol content and (B) sorbitol dehydrogenase (SDH) activity in leaves of white clover under drought stress. Vertical bars indicate ±SE of mean (n = 4). Different letters above columns indicate significant difference for comparison at a given day; the same number of asterisks above columns means significant differences for a particular treatment across days of drought stress don’t exist. LSD (*P*≤0.05). L, cv. ‘Ladino’; L+Spm, cv. ‘Ladino’ added exogenous Spm; H, cv. ‘Haifa’, H+Spm, cv. ‘Haifa’ added exogenous Spm.

As showed in Fig. 5, there was an obvious increase in sorbitol content for both white clover cultivars after 5 d of drought stress and then started to decline slightly following aggravating stress. Exogenous Spm almost have no effect on sorbitol content in leaves of both cultivars at 0 and 5 d of drought stress. However, ‘L+Spm’ or H+Spm treatment showed significantly higher sorbitol content exposed to 10 d of drought stress compared with untreated plants (‘L’ or ‘H’ treatment) (Fig. 6A). By contrast, drought stress had the most significant impact on SDH activity; thus there was a tendency that SDH activity increased gradually in both cultivars during drought stress. At 5 d of drought stress, ‘L+Spm’ or ‘H+Spm’ showed significantly greater SDH activity than ‘L’ or ‘H’, subsequently, the difference further increased at the last day of drought stress. At this time, SDH activity in ‘L+Spm’ was 2.2 times greater than that in ‘L’, and ‘H+Spm’ also showed almost 43% higher SDH activity than ‘H’ (Fig. 6B).

### Expression of dehydrins and genes

**Fig 7:**
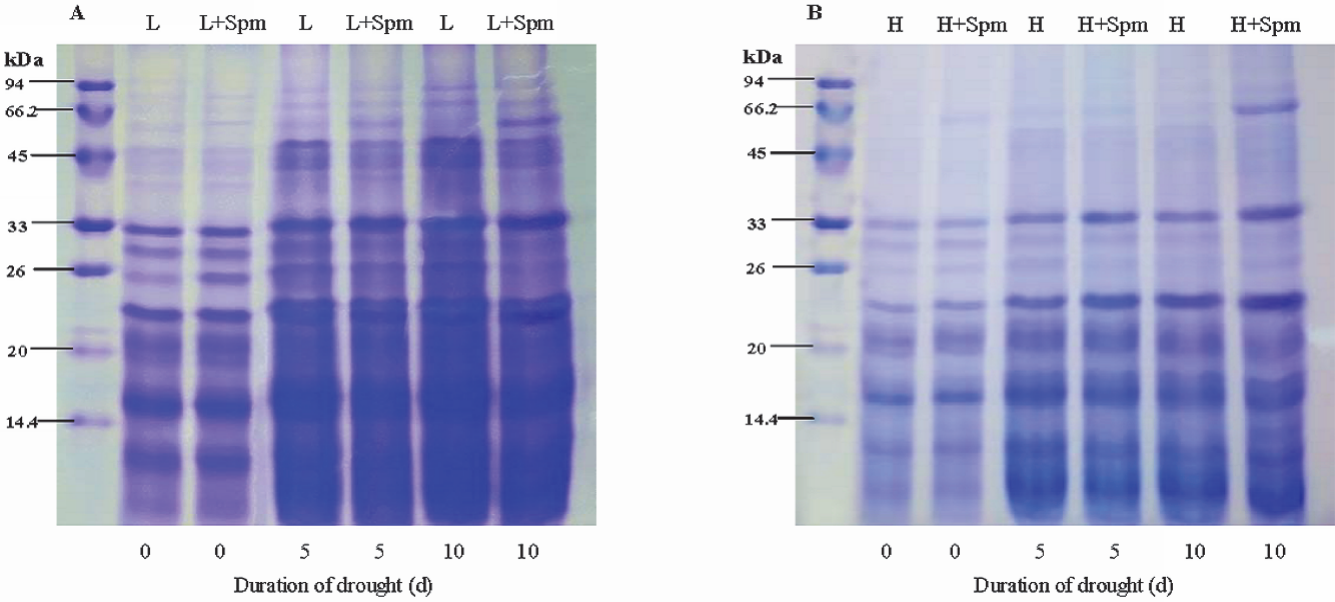
The effect of exogenous spermine (Spm) on (A) leaf heat-stable protein pattern of cv. ‘Ladino’ and (B) leaf heat-stable protein pattern of cv. ‘Haifa’ after 12% SDS-PAGE under drought stress. L, cv. ‘Ladino’; L+Spm, cv. ‘Ladino’ added exogenous Spm; H, cv. ‘Haifa’, H+Spm, cv. ‘Haifa’ added exogenous Spm.

SDS-PAGE analysis showed that heat-stable proteins accumulated in both cultivars during drought stress based on equal protein content. Some qualitative differences between protein profiles of cv. ‘Ladino’ and cv. ‘Haifa’ in the higher molecular weight range (about 45 kDa) were observed. At 10 d of drought stress, an additional protein band about 66 kDa was induced in Spm-treated plants (‘L+Spm’ and ‘H+Spm’) in response to drought stress (Fig. 7).

**Fig 8:**
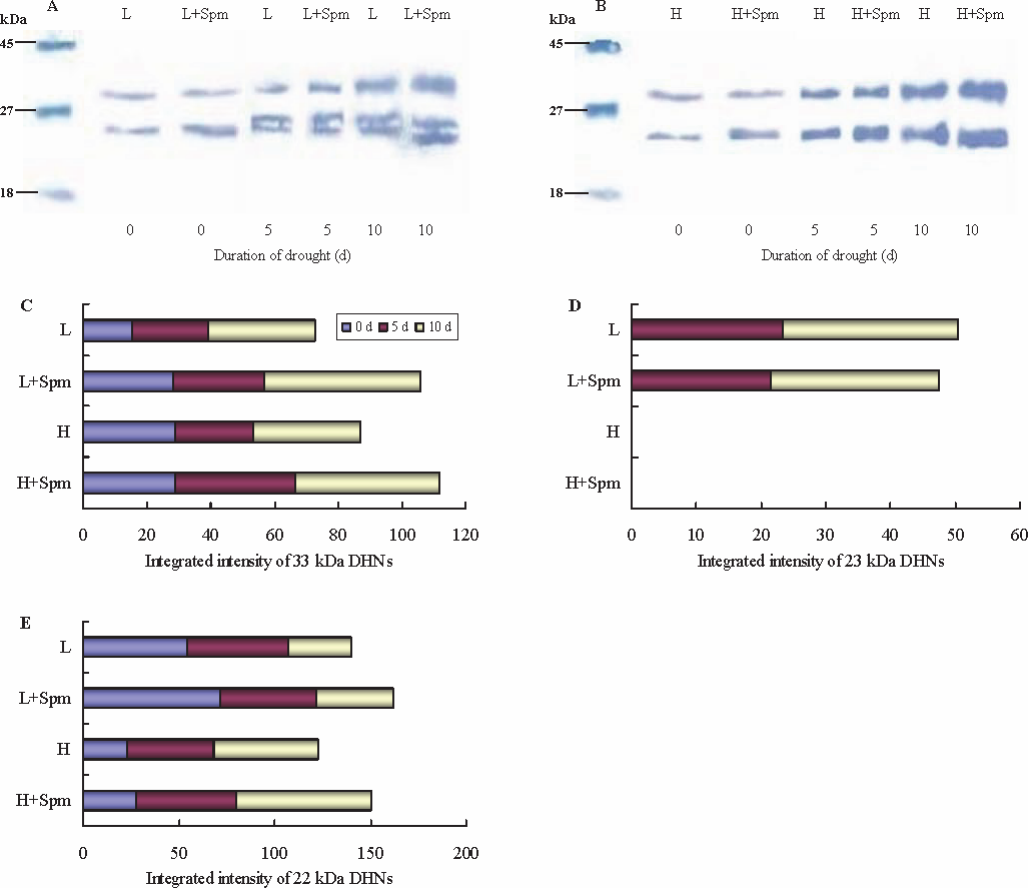
The effect of exogenous spermine (Spm) on dehydrins synthesis in white clover under drought stress. The effect of exogenous spermine (Spm) on (A) dehydrins expression in leaves of cv. ‘Ladino’, (B) dehydrins expression in leaves of cv. ‘Haifa’, (C) integrated intensity of 33 kDa dehydrin, (D) integrated intensity of 23 kDa dehydrin, (E) integrated intensity of 22 kDa dehydrin under drought stress. L, cv. ‘Ladino’; L+Spm, cv. ‘Ladino’ added exogenous Spm; H, cv. ‘Haifa’, H+Spm, cv. ‘Haifa’ added exogenous Spm.

The drought-stress inducibility of dehydrins protein synthesis in whiter clover was analysed by using western blot. Drought stress and exogenous Spm induced accumulation of dehydrins in both cultivars. There were three types of dehydrins (33, 23 and 22 kDa) in cv. ‘Ladino’ when subjected to drought stress, while only two types of dehydrins (33 and 22 kDa) were detected in cv. ‘Haifa’ (Fig. 8A,B). Spm-treated plants (‘L+Spm’ and ‘H+Spm’) exhibited obviously higher integrated intensity of 33 kDa dehydrin than non-treated plants (‘L’ and ‘H’) throughout drought stress (Fig. 8C). A 23 kDa dehydrin was induced by drought stress in cv. ‘Ladino’, but not in cv. ‘Haifa’. However, significant difference in the level of 23 kDa dehydrin was not observed between ‘L’ and ‘L+Spm’ treatment under drought stress (Fig. 8D). The change of integrated intensity of 22 kDa dehydrin in both cultivars showed a similar trend as the 33 kDa dehydrin (Fig. 8E).

**Fig 9:**
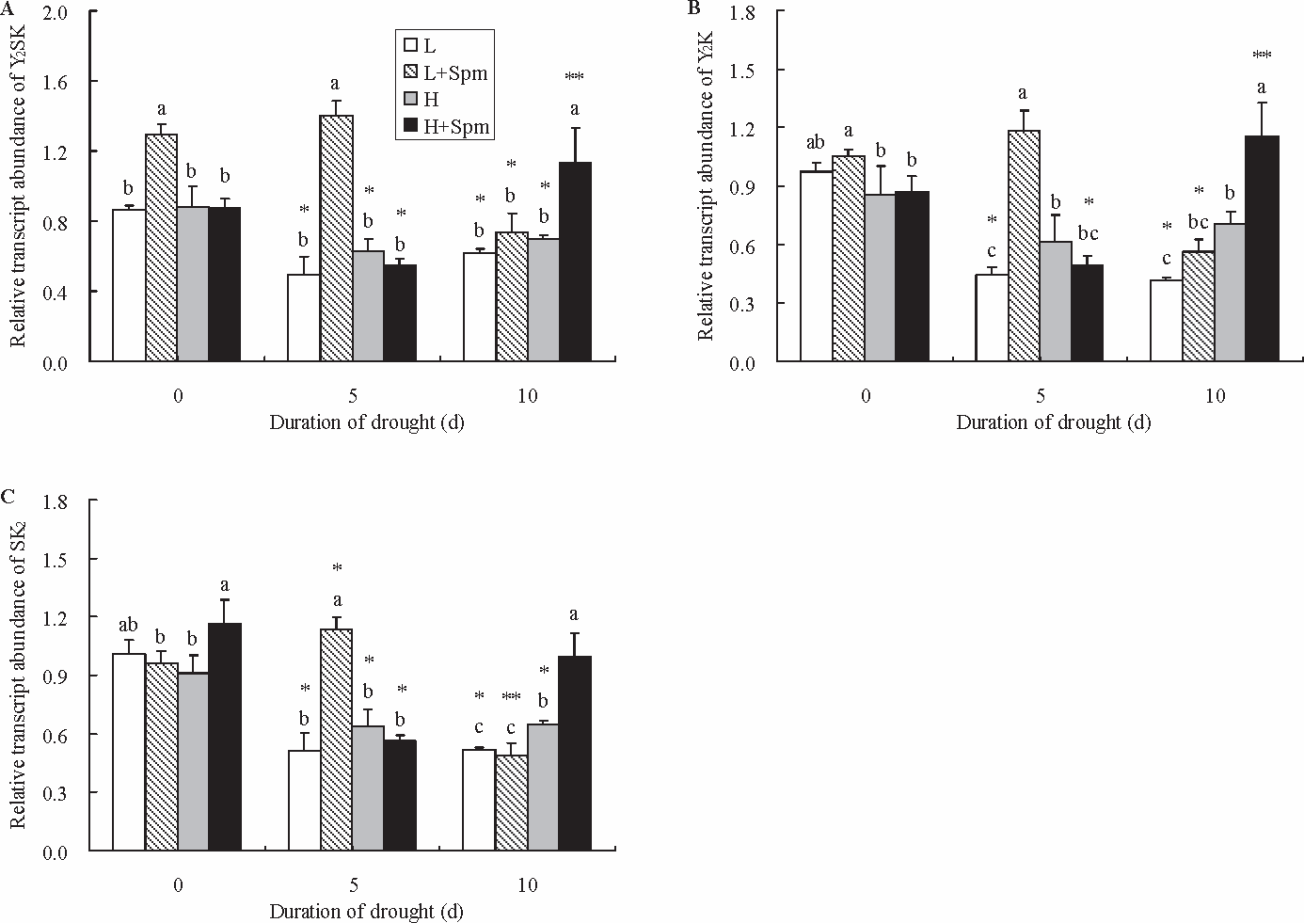
The effect of exogenous spermine (Spm) on (A) Y_2_SK gene, (B) Y_2_K gene and (C) SK_2_ gene relative transcript abundance in leaves of white clover under drought stress. Vertical bars indicate ±SE of mean (n = 4). Different letters above columns indicate significant difference for comparison at a given day; the same number of asterisks above columns means significant differences for a particular treatment across days of drought stress don’t exist. LSD (*P*≤0.05). L, cv. ‘Ladino’; L+Spm, cv. ‘Ladino’ added exogenous Spm; H, cv. ‘Haifa’, H+Spm, cv. ‘Haifa’ added exogenous Spm.

Three types of dehydrin genes were detected in leaves of white clover including Y_2_SK, Y_2_K and SK_2_ (Fig. 9). Exogenous Spm-treatment significantly up-regulated the transcript level of Y_2_SK in cv. ‘Ladino’ at 0 and 5 d of drought stress, and in particular transcript level of Y_2_SK in ‘L+Spm’ was 2.8 times higher than that in ‘L’ under 5 d of drought stress. ‘H+Spm’ also maintained significantly higher transcript level of Y_2_SK relative to ‘H’ at the end of drought stress (Fig. 9A). The transcript level of Y_2_K exhibited significant differences between Spm-treated plants (‘L+Spm’ or ‘H+Spm’) and non-treated plants (‘L’ or ‘H’) under drought stress. 170% significantly higher transcript level of Y_2_K was observed in ‘L+Spm’ as compared to that in ‘L’ treatment at 5 d of stress and after 10 d of drought stress, exogenous Spm-treatment also up-regulated the transcript level of Y_2_K in cv. ‘Haifa’ (Fig. 9B). As showed in Fig. 9C, to some degree, exogenous Spm-treatment obviously up-regulated the transcript level of SK2 in both cultivars at 5 or 10 d of drought stress, respectively.

## Discussion

The change of polyamines (PAs) level in plants is one of the most important responses to plant growth, senescence and also various environmental stress factors [43]. A large amount of researches have suggested that PAs acted as plant growth regulator or signaling molecule under drought stress [43–45] and exogenously applied Spm could improve drought tolerance of plants by inhibiting lipid peroxidation, increasing water use efficiency and modulating plant metabolism [27,46]. Farooq et al. [22] observed that exogenous Spm treatment elevated the RWC of rice under osmotic stress, which was consistent with the results in this study. Our present results also showed that exogenous Spm was effective on alleviating lipid peroxidation (the reduction in MDA content and EL level in Spm-treated plants) and degradation of chlorophyll caused by drought stress in both white clover cultivars. This further confirmed the positive effects of Spm on improving drought tolerance of plants.

Water deficit particularly leads to increase the accumulation of water-soluble carbohydrates (WSC) including sucrose, fructose, and glucose in plants. Sucrose is critical for energy source and plays an important role in maintaining hydrophilic structures of proteins and stabilization of cell membrane when plant is subject to drought stress [47]. Fructose and glucose under drought stress not only has been proved to be important compatible osmolytes involved in decreasing osmotic potential but also could function as raw materials of carbon metabolism, or as initiate signal transduction factors in stress signaling pathway [48,49]. Al Hakimi et al. [50] and Kaur et al. [48] presumed that the accumulation of soluble sugars in wheat was one of the main features to improve drought tolerance. Previous reports have shown that PAs was concerned with carbohydrate metabolism under multiple abiotic stresses. Spd effectively alleviated chill-induced metabolic disturbance of carbohydrate in leaves of spinach [51]. Exogenous Put improved drought tolerance of wheat by increasing accumulation of soluble sugar in leaves [52]. In the current study, Spm application led to accumulation of high levels of WSC, sucrose and fructose in both white clover cultivars under drought stress. Exogenous Spm also significantly elevated glucose content in leaves of drought-resistant cv. ‘Haifa’, but had no effect on drought-susceptible cv. ‘Ladino’. It suggests that there are differences between drought-resistant and-susceptible white clover cultivars related to Spm regulation of WSC accumulation. Sucrose synthetase (SS) and sucrose phosphate synthase (SPS) regulated sucrose metabolism in plants [53]. SS plays a central role in secondary metabolism of sucrose. When plants are subjected to chilling, drought, and salt stress, SS will catalyze the synthesis of sucrose by using glucose and fructose as substrates. However, this catalytic process is reversible, which means SS also could decompose sucrose into glucose and fructose [54,55]. The activity of SS changed with duration of drought stress or degree of water deficit because of the function of SS in catalyzing both sucrose synthesis and degradation [56,57]. The difference between SS and SPS is that SPS is located in the cytoplasm, only catalyzes sucrose synthesis and is involved in the most critical synthetic route of sucrose in higher plants. Additionally, SPS is widely implicated in many stress response mechanisms [58]. Yang et al. [59] and Fresneau et al. [58] found that drought induced an increase in SPS activity of rice and wheat. Under drought stress, exogenous Spm significantly increased SPS activity, but decreased SS activity in both white clover cultivars. This agrees with the early report of Kaur et al [48]: higher sucrose content along with a higher SPS activity and a lower SS activity could be responsible for drought tolerance in wheat. Altogether, it may suggest that the increase of Spm-induced sucrose content depends on SPS under drought stress, while SS has more possibilities to be involved in the balance between sucrose synthesis and degradation.

Sorbitol serves as the major polyol, and is also a unique translocated form of carbon in plants. It has been shown that drought stress improved the accumulation of sorbitol which contributed to drought tolerance of plants [6,60,61]. Li et al. [61] reported that the overexpression of two sorbitol transporter genes in *Arabidopsis* induced the increase of sorbitol content associated with acquiring strong drought tolerance. From currently available data, we found that sorbitol content increased significantly under drought stress in both Spm-treated and Non-treated plants, which indicated that white clover was just like other plants through increasing sorbitol content to cope with drought stress. Furthermore, exogenous Spm improved the accumulation of sorbitol in both cv. ‘Ladino’ and ‘Haifa’ at the last stage of drought stress, thereby influencing osmoregulation capacity of white clover to maintain cell turgor. SDH provides a way for plans to convert sorbitol into fructose without using ATP [62]. According to the reports of Bianco et al. [10] and Li and Li [63], the increase of SDH activity was a key factor for catabolism of sorbitol in response to drought stress. The *sdh*-mutants of *Arabidopsis* with inhibition of SDH activity exhibited less dry weight and root length compared to wild-type under supply of exogenous sorbitol condition [64]. Therefore, it can be deduced that increased SDH activity induced by exogenous Spm in both white clover cultivars may play a part in converting sorbitol into fructose to keep balance for metabolism of sorbitol.

Under abiotic stress, exogenous ABA-, cytokinin- or proline-induced increase in the expression of dehydrins proves that various phytohormones or physiological activators are associated with regulation of dehydrins in plants [65–67]. Previous studies have indicated that Spm and dehydrins had the same functions of scavenging reactive oxygen species (ROS) and maintaining the structure of membrane [27,68]. Although it was confirmed that Spm and dehydrins both could enhance the drought tolerance of plants [11,22,69], the relationship between Spm and dehydrins has not been fully elucidated. Spm, one of the most active PAs, was suggested to acts as a signaling regulator during stress. Spm regulated the generation of nitric oxide (NO) signal in *Arabidopsis thaliana* seedlings [70] and also interacted with ethylene (ETH) or ABA to improve drought tolerance of plants [71–73]. In blueberry, changes in dehydrins expression depended on endogenous ABA levels and drought intensity [74]. Vaseva et al. [40] reported that drought-resistant white clover cultivar accumulated more heat-stable proteins and dehydrins along with higher genes transcript levels encoding dehydrins than the sensitive one when they were subjected to drought stress. In *Dendrobium candidum*, the expressions of heat-stable proteins and dehydrins induced by ABA have positive effects on dehydration and freezing tolerance [11]. The similar results were carried out in *Solanum s*pecies about functions of heat-stable proteins and dehydrins [75]. We noticed exogenous Spm induced an additional heat-stable protein band about 66 kDa after 10 d of drought stress in both cultivars. Moreover, Spm significantly enhanced the accumulation of dehydrins (22 and 33 kDa) and the transcript level of three genes encoding dehydrins in both cultivars during drought stress. These suggest that Spm is concerned with regulation of dehydrins in white clover. In addition, the data in this study also showed that drought-susceptible white clover cv. ‘Ladino’ accumulated the special dehydrin (23 kDa) after 5 d of drought, but it wasn’t observed in drought-resistant cv. ‘Haifa’. Spm-induced transcript levels of dehydrin genes increased in cv. ‘Ladino’ earlier than that in cv. ‘Haifa’. Thus, it could be revealed that the synthesis of dehydrins and genes expression encoding dehydrins regulated by exogenously applied Spm are correlated with white clover cultivars with different drought tolerance. The studies of Blackman et al. [76] and Walters et al. [77] suggested that interactions between sugars and heat-stable proteins improved the dehydration tolerance of plants. However, the correlation between carbohydrate and dehydrins related to Spm regulation can’t be fully elucidated in this study and deserves further investigation.
